# Investigation of single nucleotide polymorphisms in differentially expressed genes and proteins reveals the genetic basis of skeletal muscle growth differences between Tibetan and Large White pigs

**DOI:** 10.5713/ab.24.0135

**Published:** 2024-06-25

**Authors:** Heli Xiong, Yan Zhang, Zhiyong Zhao

**Affiliations:** 1 Animal Nutrition and Swine Institute, Yunnan Academy of Animal Husbandry and Veterinary Sciences, Kunming 650224, China

**Keywords:** Differentially Expressed Genes, Differentially Expressed Proteins, Large White Pigs, Single Nucleotide Polymorphisms (SNPs), Skeletal Muscle Growth, Tibetan Pigs

## Abstract

**Objective:**

Skeletal muscle growth is an important economic trait for meat production, with notable differences between Tibetan pigs (TIBPs, a slow-growing breed) and Large White pigs (LWPs, a fast-growing breed). However, the genetic underpinnings of this disparity remain unclear.

**Methods:**

In the current study, we integrated differentially expressed genes (DEGs) and proteins (DEPs) from 60-day-old embryonic muscle tissue, along with whole-genome single nucleotide polymorphisms (SNPs) displaying absolute allele frequency differences (ΔAF) of 0.5 or more between the TIBP and LWP breeds, to unravel the genetic factors influencing skeletal muscle growth.

**Results:**

Our analysis revealed 3,499 DEGs and 628 DEPs with SNPs having a ΔAF equal to or greater than 0.5. Further functional analysis identified 145 DEGs and 23 DEPs involved in biological processes related to skeletal muscle development, and 22 DEGs and 3 DEPs implicated in the mechanistic target of rapamycin kinase signaling pathway, which is known for positively regulating protein synthesis. Among these genes, several DEGs and DEPs, enriched with TIPB-specific SNPs in regulatory or/and coding regions, showed marked ΔAF between the TIBP and LWP breeds, including *MYF5*, *MYOF*, *ASB2*, *PDE9A*, *SDC1*, *PDGFRA*, *MYOM2*, *ACVR1*, *ZIC3*, *COL11A1*, *TGFBR1*, *EDNRA*, *TGFB2*, *PDE4D*, *PGAM2*, *GRK2*, *SCN4B*, *CACNA1S*, *MYL4*, *IGF1*, and *FOXO1*. Additionally, genes such as *CAPN3*, *MYOM2*, and *PGAM2*, identified as both DEPs and DEGs related to skeletal muscle development, contained multiple TIBP-specific and LWP-predominant SNPs in regulatory and/or coding regions, underscoring significant ΔAF differences between the two breeds.

**Conclusion:**

This comprehensive investigation of SNPs in DEGs and DEPs identified a significant number of SNPs and genes related to skeletal muscle development during the prenatal stage. These findings not only shed light on potential causal genes for muscle divergence between the TIBP and LWP breeds but also offer valuable insights for pig breeding strategies aimed at enhancing meat production.

## INTRODUCTION

Tibetan pigs (TIBPs), a native mini-type breed from China, are renowned for their high meat quality and favorable status in premium markets. Despite their popularity, these pigs are characterized by lower meat production, attributable to their smaller body size and slower growth rate than other breeds. Typically, a TIBP weighs approximately 25 kg at 1 year of age, starkly contrasting with Large White pigs (LWPs), which can reach a similar weight in just 2 months and often attain approximately 150 kg at around 8 months [1]. However, the genetic basis for the lower body size and slower growth rate in the TIBP breed remains elusive.

The postnatal growth rate and growth potential of pigs are predominantly determined by the number of muscle fibers that develop during embryogenesis [2]. It has been observed that littermates with a higher count of muscle fibers exhibit faster and more efficient growth than those with fewer fibers. Notably, fast-growing pig breeds generally possess a greater number of muscle fibers than slow-growing breeds [2]. For instance, LWPs, when compared with mini-pigs, demonstrate that variations in muscle size can be attributed directly to differences in myofiber number [3]. Similarly, a comparison between fast-growing Landrace pigs and slow-growing Lantang pigs revealed that the former typically have a higher muscle fiber count than the latter [4]. The development of muscle fibers is established through two primary phases of fiber generation before birth. The initial phase occurs between 35 and 60 days post-coitus (dpc), followed by a second phase between 54 and 90 dpc. After these two stages of myogenesis, the total muscle fiber count is established and remains constant [4,5]. Consequently, genes that influence myoblast differentiation and muscle fiber formation during embryogenesis are crucial in determining postnatal growth and overall body size in pigs.

The formation of muscle fibers is a complex process orchestrated by the coordinated expression of various transcription factors. Central to this regulation are myogenic regulatory factors (MRFs), including *MYOD*, *MYOG*, *MYF5*, *MRF4*, and the myocyte enhancer factor 2 (MEF2) family, specifically *MEF2A*, MEF2B, *MEF2C*, and *MEF2D* [6]. *MYOD* and *MYF5* play pivotal roles in guiding embryonic mesodermal progenitor cells to differentiate into myoblasts, whereas *MYOG* and either *MYOD* or *MRF4* (*MYF6*) are instrumental in the subsequent transformation of myoblasts into mature myocytes. This process involves the synergistic action of *MyoD* and MEF2 family members, which activate the transcription of key skeletal muscle genes, such as M-creatine kinase, myosin heavy chain, and desmin [6–9]. Muscle growth is achieved when the rate of protein synthesis exceeds that of degradation within muscle tissue. Two major signaling pathways, the mechanistic target of rapamycin kinase (mTOR) signaling pathway and myostatin-Smad2/3 pathway, function as positive and negative regulators of muscle growth, respectively [10]. In particular, the mTOR pathway is vital for regulating protein synthesis and is influenced by upstream activators, such as growth factors (e.g., IGF1 and insulin), which act through the PI3K-Akt cascade and various amino acids via Rag GTPases [11]. These intricate molecular interactions underscore the complexity and precision of muscle growth regulation.

Comparative studies examining prenatal muscle tissue through transcriptome or proteome profiling between breeds exhibiting divergent growth characteristics offer valuable insights into developmental differences in muscle tissue and collaborative regulation of myofiber formation by numerous genes [4,12–14]. The integration of genomic variations identified through whole-genome sequencing with differentially expressed genes (DEGs) and proteins (DEPs) observed in transcriptome or proteome analyses between breeds with extreme phenotypes enhances our understanding of the genetic basis underlying these divergent characteristics and aids in identifying causal genes [15]. Shang et al [16] reported the transcriptome and proteome profiles in 60-day-old embryonic longissimus dorsi muscle tissues from TIBP and LWP breeds, indicating that numerous genes showed significant differeces in mRNA and protein expression level between these two breeds. However, their study had limitations in comprehensively elucidating the genetic differences of those genes exhibiting significant different expression level. A genome-wide single nucleotide polymorphism (SNP) will help reveral the genetic variations that may contribute to expression differences between the two breeds.

Therefore, in this study, we investigated SNPs present in DEGs and DEPs in 60-day-old embryonic muscle tissues from the TIBP and LWP breeds. This was achieved by transcriptome and proteome data from prior research with whole-genome SNPs that exhibited absolute allele frequency differences (ΔAF) of 0.5 or greater between the TIBP and LWP breeds, as identified in our previous study. We identified a significant number of TIBP- and LWP-specific and predominant SNPs in the regulatory and coding regions with marked ΔAF between the TIBP and LWP breeds, which were present in an array of genes implicated in various aspects of striated muscle biology, including cell differentiation (*MYF5*, *MYOF*, and *ASB2*), hypertrophy (*PDE9A*), cell development (*SDC1*, *PDGFRA*, *MYOM2*, *ACVR1*, and *ZIC3)*, tissue development (*COL11A1*, *TGFBR1*, *EDNRA*, and *TGFB2*), muscle contraction (*PDE4D*, *PGAM2*, *GRK2*, and *SCN4B*), muscle adaptation (*CACNA1S* and *MYL4*), and the protein synthesis signaling pathway (*IGF1* and *FOXO1*). These findings point to a critical link between these genes and the observed differences in skeletal muscle growth between the TIBP and LWP breeds, suggesting their potential role in breed-specific muscle development.

## MATERIALS AND METHODS

All the experimental procedures were reported in accordance with ARRIVE guidelines and approved by the Animal Care and Use Committee of Yunnan Academy of Animal Husbandry and Veterinary Sciences. The care and use of animals were fully in compliance with local animal welfare laws, guidelines, and policies.

### Data utilization

For this study, we used whole-genome SNPs that displayed ΔAF between the TIBP and LWP breeds. These SNPs were derived from our previous study, which involved whole-genome sequencing of 44 TIBP and 29 LWP individuals. This dataset produced 16.47 billion raw reads, with a mean depth of 9.13× per individual and average genome coverage of 98.96%. A total of 21,594,848 and 15,210,134 SNPs were identified for TIBP and LWP, respectively. In total, 21,767,938 SNPs were obtained from the 73 individuals for subsequent analysis. SNPs were categorized based on their specificity and predominance in each breed. SNPs with allele frequencies of 0.5 or higher in TIBP and nonexistent in LWP were classified as TIBP-specific. Those with allele frequencies greater than 0.5 in TIBP and less than 0.5 in LWP, accompanied by an absolute ΔAF equal to or exceeding 0.5, were termed TIBP-predominant SNPs. Conversely, SNPs with allele frequencies of 0.5 or higher in LWP and nonexistent in TIBP were designated LWP-specific. SNPs with allele frequencies greater than 0.5 in LWP, less than 0.5 in TIBP, and ΔAF between LWP and TIBP of 0.5 or more were labeled as LWP-predominant SNPs.

Shang et al [16] detected the transcriptome and preteome profiles in longissimus dorsi muscle tissues of nine embryos from two pregnant TIBP and LWBP sows at 60 days after insemination. They identified 3,858 DEGs and 830 DEPs, respectively, between the TIBP and LWP breeds. In this study, we integrated the identified 3,858 DEGs and 830 DEPs into our analysis. This integration aimed to enrich our understanding of the genetic variations of genes involved in skeletal muscle development divergenece between these two pig breeds.

### Identification of TIBP- and LWP-specific and predominant SNPs in DEGs and DEPs

In our study, we meticulously annotated specific and predominant SNPs for TIBP and LWP breeds and assigned them to corresponding genes, referred to as “candidate genes.” We then conducted a comprehensive comparison of the candidate genes, DEGs, and DEPs. This process enabled us to identify genes that overlapped between the candidate genes and DEGs and DEPs. To visualize the overlap and relationships among these genes, we used OmicStudio tools (available at https://www.omicstudio.cn/tool), creating a Venn diagram for an intuitive representation of our findings. Additionally, we used custom-developed Python scripts to extract SNPs within overlapping DEGs and DEPs.

### Functional enrichment analysis of overlapping DEGs

To delve deeper into the functional implications of the overlapping DEGs, we conducted a gene ontology (GO) enrichment analysis using g:Profiler tools (available at https://biit.cs.ut.ee/gprofiler/orth) [17]. In this analysis, we set organism to "*Sus scrofa*", statistical domain scope set to "only annotated genes", significance threshold set to "Benjamini-Hochberg FDR", and the user threshold set to "0.05".

Subsequently, we performed Kyoto encyclopedia of genes and genomes (KEGG) pathway analysis using the DAVID database (https://david.ncifcrf.gov/tools.jsp) [18], a widely recognized platform for high-throughput gene function analysis. Again, the organism was specified as "*Sus scrofa*," and we adhered to the default settings for the other parameters. This approach enabled us to identify potential genes involved in skeletal muscle development, as indicated by their enrichment in the relevant GO terms and KEGG pathways.

We investigated the allele frequencies of SNPs within these identified genes to further refine our insights. This was achieved using in-house custom-developed scripts, which allowed us to identify specific genetic variations that potentially drive skeletal muscle development.

### Functional enrichment analysis of overlapping DEPs

We conducted a GO enrichment analysis to elucidate the functional roles of the overlapping DEPs in skeletal muscle growth. This was achieved using the g:Profiler tool (available at https://biit.cs.ut.ee/gprofiler/orth) [17]; the organism was set to "Sus scrofa", the statistical domain scope was set to "only annotated genes", the significance threshold was set to "Benjamini-Hochberg FDR", and the user threshold was set to "0.05".

Furthermore, we performed a KEGG pathway analysis using the DAVID database (https://david.ncifcrf.gov/tools.jsp) [18]. For this analysis, the organism was again set to "*Sus scrofa*", and the default settings were maintained for other parameters. This approach allowed us to identify DEPs that were significantly enriched in GO terms and KEGG pathways pertinent to skeletal muscle development, suggesting their potential role in this process.

We investigated the allele frequencies of SNPs contained within them to deepen our understanding of these DEPs. This task was accomplished using custom-developed scripts, enabling a detailed examination of genetic variations that potentially influence skeletal muscle development.

## RESULTS

### TIBP- and LWP-specific and predominant SNPs in DEGs and DEPs

**Figure 1:**
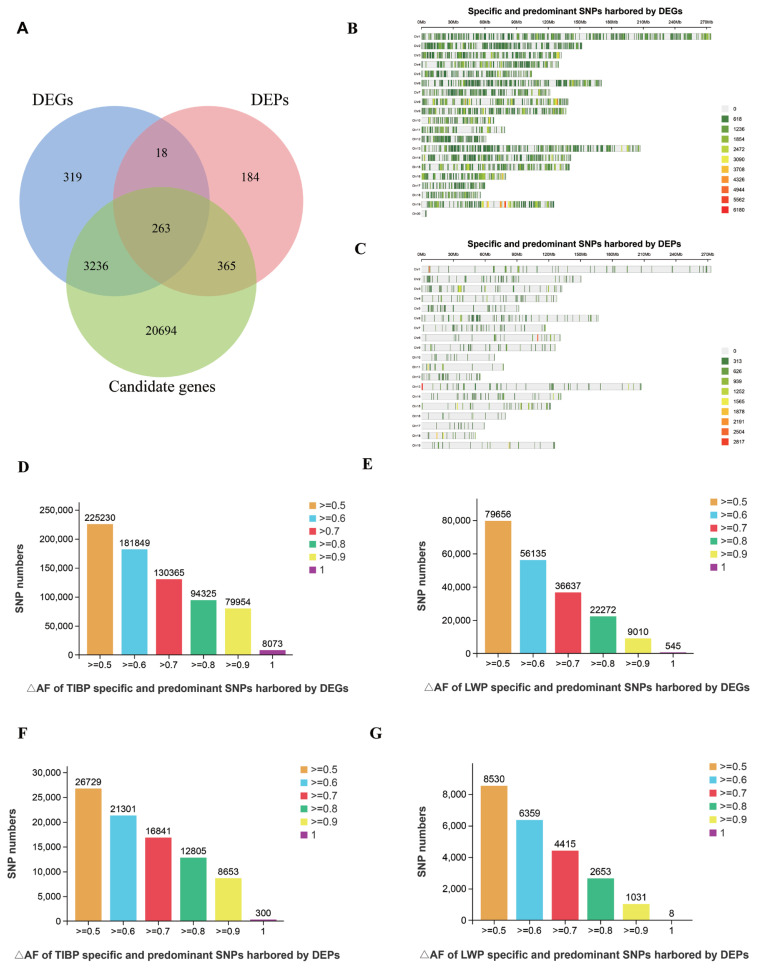
Summary of TIBP- and LWP-specific and predominant SNPs. (A) A Venn diagram illustrating the overlap of genes among candidate genes, DEGs, and DEPs; (B) and (C) the chromosomal distribution of TIBP- and LWP-specific and predominant SNPs found within DEGs and DEPs; and (D)–(G) the counts of SNPs with ΔAF ranging from 0.5 to 1.0 for TIBP- and LWP-specific and predominant SNPs harbored by DEGs and DEPs. TIBP, Tibetan pig; LWP, Large White pig; SNPs, single nucleotide polymorphism; DEGs, differentially expressed genes; DEPs, differentially expressed proteins; AF, allele frequency differences.

In our previouly study, 2,893,106 and 813,310 specific and predominant SNPs in the TIBP and LWP populations were identified and annotated to 24,560 genes. In this study, we referred to these annotated genes as “candidate genes”. Shang et al [16] reported a significant number of DEGs and DEPs, specifically 3,858 DEGs and 830 DEPs, in the longissimus dorsi muscle tissues from the TIBP and LWP breeds at 60 days of embryonic development. Upon comparing these candidate genes with DEGs and DEPs, we discovered that 3,499 DEGs and 628 DEPs overlapped with candidate genes (Figure 1A). This overlap represented 91.27% of the total DEGs and 75.67% of the total DEPs, indicating that a major part of these genes and proteins contained TIBP- and LWP-specific and predominant SNPs. Specifically, 3,499 DEGs contained 924,051 specific and predominant SNPs, including 270,189 TIBP-specific and 449,608 predominant SNPs, along with 190,210 LWP-specific and 14,045 predominant SNPs. Furthermore, 628 DEPs contained 109,625 specific and predominant SNPs, comprising 32,938 TIBP-specific and 53,692 predominant SNPs, in addition to 1,983 LWP-specific and 21,014 predominant SNPs. We observed that these specific and predominant SNPs were distributed across all chromosomes (Figure 1B and 1C). The ΔAF values of these SNPs ranged from 0.5 to 1.0, with the majority falling within the 0.5 to 0.6 range (illustrated in Figure 1D–1G). Furthermore, we identified 263 genes shared among the DEGs, DEPs, and candidate genes (Figure 1A).

### Investigation of SNPs in DEGs related to skeletal muscle development

We conducted a GO enrichment analysis of the overlapping DEGs to investigate SNPs present in the DEGs associated with skeletal muscle growth. This analysis revealed that 3,499 overlapping genes were significantly enriched in 1,481 biological processes, 246 cell components, and 151 molecular functions (Supplementary Table S1). Focusing specifically on skeletal muscle development, a type of striated muscle, we focused on the biological processes relevant to this area. We identified seven striated muscle biological process-related GO terms, encompassing striated muscle cell differentiation, development, tissue development, contraction, adaptation, hypertrophy, and negative regulation of muscle contraction. Among these categories, 145 genes were identified, including a significant number of SNPs: 5,983 TIBP-specific and 16,894 predominant SNPs, along with 433 LWP-specific and 7,072 predominant SNPs (Supplementary Table S2). These SNPs were classified into 13 distinct types, including missense, synonymous, 5′ prime untranslated regions (UTR), and 3′ prime UTR variations. The majority of these SNP were variations in the intronic region, accounting for 66.2% of the total. Supplementary Table S3 presents a detailed breakdown of each SNP type, providing a thorough understanding of the SNP landscape in DEGs associated with skeletal muscle development.

We identified 145 genes, including two members of the MRF family (*MYF5* and *MYF6*) and three main members of the MEF family (*MEF2A*, *MEF2C*, and *MEF2D*), which are key to skeletal muscle development. Additionally, genes such as *MSTN*, which act as negative regulators of muscle development, were identified. SNPs located in the exonic, splice, and regulatory regions, including synonymous, missense, splice region, 3′ prime UTR, and 5′ prime UTR, as well as downstream and upstream variations, can significantly influence gene structure and function. Breed-specific SNPs are crucial in defining breed-specific traits. Therefore, we focused on genes containing TIBP-specific SNPs in regulatory and/or coding regions, especially those with allele frequencies exceeding 0.80. This led to the discovery of several genes harboring multiple regulatory and/or coding region TIBP-specific SNPs with allele frequencies greater than 0.8. Table 1 presents detailed information on these SNPs. The genes identified include *MYF5*, *MYOF*, and *ASB2* (implicated in striated muscle cell differentiation); *PDE9A* (associated with striated muscle hypertrophy); *SDC1*, *PDGFRA*, *MYOM2*, *ACVR1*, and *ZIC3* (involved in striated muscle cell development); *COL11A1*, *TGFBR1*, *EDNRA*, and *TGFB2* (contributing to striated muscle tissue development); *PDE4D*, *PGAM2*, *GRK2*, and *SCN4B* (related to striated muscle contraction); and *CACNA1S* and *MYL4* (associated with striated muscle adaptation).

KEGG pathway analysis revealed significant enrichment of over-represented genes across 118 distinct pathways, as detailed in Supplementary Table S4. These pathways encompass critical biological processes, including energy production (such as metabolic pathway, carbon metabolism, citrate cycle, and pyruvate metabolism), fat metabolism (fatty acid metabolism and fatty acid degradation), adipocyte differentiation (PPAR signaling pathway), actin dynamics (regulation of actin cytoskeleton), and amino acid metabolism (biosynthesis of amino acids, valine, leucine, and isoleucine degradation, arginine biosynthesis, arginine, and proline metabolism). Several signaling pathways, including insulin [11], AMPK [19], FoxO [10], PI3K-Akt [20], ERBB [21], HIF-1 [22], MAPK [23], and P53 signaling pathways [24], play roles in various biological processes, notably in protein synthesis and degradation via regulation of the mTOR signaling pathway. Consequently, we identified 22 genes involved in the mTOR signaling pathway. This set included genes such as *IGF1*, *INSR*, *IRS3*, *PIK3R2*, *SGK1*, *AKT2*, *SKP2*, *MDM2*, *RPS6KB2*, *RHEB*, *EIF4EBP1*, *SHC1*, *RAF1*, *FOXO1*, *MAP2K2*, *MKNK2*, *STK11*, *AKT1S1*, *RB1CC1*, *ULK1*, *ERBB2*, and *GAB1*, which collectively harbor 3,915 specific and predominant SNPs (Supplementary Table S5). Supplementary Table S6 presents detailed classifications of these SNP types. Further analysis of allele frequencies revealed that genes such as *IGF1*, *FOXO1*, and *EIF4EBP1* possess functionally significant TIBP-specific SNPs with allele frequencies exceeding 0.80, as shown in Table 2. Notably, *IGF1* includes several SNP types in regulatory regions, including the 5′ prime UTR, upstream, 3′ prime UTR, and downstream regions.

### Investigation of SNPs in DEPs related to skeletal muscle development

Our GO enrichment analysis of the 628 overlapping DEPs identified significant enrichment across 270 biological processes, 177 cell components, and 95 molecular function terms, as detailed in Supplementary Table S7. This analysis revealed four biological processes related to the GO terms associated with striated muscle, including striated muscle cell development, hypertrophy, contraction, and adaptation. Within these categories, 23 genes were identified. These genes collectively harbored 3,114 specific and predominant SNPs, which are cataloged in Supplementary Table S8. These SNPs spanned 12 distinct SNP types, with a notable majority being intergenic region variations, accounting for 57.1%, as outlined in Supplementary Table S9. An examination of allele frequencies highlighted several significant findings: one upstream TIBP-specific SNP in the *MYL4* genes with 0.93 allele frequency, a splice region variant and intron TIBP-specific SNP in the *MYOM2* gene with an allele frequency of 0.86, and two 5′ prime UTR plus one 5′ prime UTR premature start codon gain TIBP-specific SNPs in *PGAM2*, averaging an allele frequency of 0.84.

KEGG pathway analysis revealed significant enrichment in 51 over-represented pathways, as detailed in Supplementary Table S10. These pathways encompass a broad range of biological functions, including energy production (e.g., metabolic pathways, carbon metabolism, and citrate cycle), fat metabolism (such as fatty acid metabolism and fatty acid degradation), and amino acid metabolism (including biosynthesis of amino acids, valine, leucine, and isoleucine degradation, arginine biosynthesis, arginine, and proline metabolism). Furthermore, pathways integral to protein synthesis and degradation were highlighted, notably the insulin and HIF-1 signaling pathways. Within the realm of these pathways, genes such as *EIF4EBP1*, *EIF4E2*, and *EIF4E* have been identified as being involved in the mTOR signaling pathway.

### SNP analysis of shared genes in DEGs and DEPs linked to skeletal muscle development

In our study, we delved deeper into the shared genes found between DEGs and DEPs that play roles in biological processes associated with striated muscle development. The results revealed that 18 genes were overlapped. These are *CASQ1*, *MYOM2*, *TPM1*, *ACTA1*, *CAPN3*, *NEB*, and *MYOZ1* (involved in striated muscle cell development); *TCAP*, *RAP1GDS1*, *ATP2B4*, *LMCD1*, and *TWF1* (associated with striated muscle hypertrophy); and *TNNC2*, *MYL4*, *ATP2A1*, *PGAM2*, *HOMER1*, and *STAC3* (related to striated muscle contraction). A total of 2,930 specific and pedominant SNPs were identified within these genes (Supplementary Table S11). Notably, several of these genes harbored functionally significant specific and predominant SNPs, with ΔAF exceeding 0.80. These included genes such as *TPM1*, *CAPN3*, *RAP1GDS1*, *MYOZ1*, *MYOM2*, and *PGAM2*, as detailed in Table 3. Specifically, the *CAPN3* gene contained four synonymous and three 5′ prime UTR TIBP-predominant SNPs; the *MYOM2* gene had five synonymous and one splice region variant and intron TIBP-predominant SNPs; and the *PGAM2* gene included one 5′ prime UTR premature start codon gain, two 5′ prime UTR TIBP-specific SNPs, four 5′ prime UTR SNPs, one 5′ prime UTR premature start codon gain and one synonymous LWP-predominant SNP.

Additionally, *EIF4EBP1* emerged as a shared gene among both DEGs and DEPs, as identified in our KEGG pathway analysis. The *EIF4EBP1* gene was found to contain 22 TIBP-specific SNPs, comprising 20 intronic variations and two upstream variations. Within the TIBP population, the SNPs exhibited an average allele frequency of 0.76, highlighting their genetic distinctiveness and potential functional impact in this breed.

## DISCUSSION

Our study presents a novel approach for identifying genes that influence growth rates by examining the frequency of genomic SNPs within DEGs and DEPs. This method was applied to two genetically divergent pig breeds at critical stages of growth. Our findings, which revealed a substantial number of DEGs and DEPs with significant SNP frequency differences between the TIBP and LWP breeds, particularly in genes associated with muscle development, highlight the genetic factors that contribute to variations in muscle growth traits. We focused on analyzing the SNP frequency in DEGs and DEPs in 60-day-old embryonic muscle tissues from the TIBP and LWP breeds. This developmental stage is crucial as it marks the formation of primary muscle fibers and lays the foundation for postnatal growth [4]. The genes identified at this stage may play a pivotal role in determining the differences in myofiber formation between breeds. In the context of population genetics, disparities in traits between breeds are fundamentally attributed to variations in allele frequencies. Therefore, the detection of genomic SNP frequencies in DEGs and DEPs offers invaluable insights and aids in the identification of potential causative genes that govern muscle growth and development.

### Potentially crucial DEGs related to skeletal muscle growth

The MRF and MEF families are pivotal in muscle cell differentiation and development and play central roles in the regulation of myogenesis [9]. Notably, in our study, two members of the MRF family, *MYF5* and *MYF6*, and three principal members of the MEF family, *MEF2A*, *MEF2C*, and *MEF2D*, emerged as DEGs. Intriguingly, these genes harbored multiple TIBP- and LWP-specific and predominant SNPs. Moreover, *MSTN*, a member of the transforming growth factor beta (TGF-β) superfamily produced by skeletal muscle, acts as a key inhibitor of muscle growth. The occurrence of myostatin mutations across various mammalian species, which lead to muscle hypertrophy, underscores its vital role in modulating muscle development and size. Our study further identified *MSTN* as a DEG containing several TIBP- and LWP-specific and predominant SNPs. This finding aligns with previous research, underscoring the significance of these genes in muscle fiber formation and potentially elucidating the observed disparities in muscle growth rates between the TIBP and LWP breeds.

The discovery of SNPs within the regulatory and/or coding regions of DEGs and DEPs associated with muscle growth is a key achievement of our study. Particularly noteworthy were the TIBP- and LWP-specific and predominant SNPs that exhibited marked ΔAF between the TIBP and LWP breeds. These SNPs potentially contribute to the slower muscle growth observed in TIBP than in LWP. SNPs located upstream, 3′ prime UTR, 5′ prime UTR, downstream, and splice regions have the potential to modulate gene expression levels and patterns [25]. Their influences on post-transcriptional regulation, particularly affecting mRNA stability and translation efficiency, may play a crucial role in driving phenotypic disparities in muscle development observed between the TIBP and LWP breeds. Furthermore, SNPs in coding regions, such as missense variations, can alter amino acid sequences [26], thereby influencing the structure and functionality of proteins vital for muscle development. Even synonymous SNPs, traditionally thought to be silent, can affect gene expression and protein folding, as indicated in a recent study [27].

In this study, we observed that *GRK2*, *ASB2*, *PDGFRA*, *ACVR1*, *PGAM2*, *IGF1*, and *FOXO1* were DEGs containing several TIBP-specific SNPs in regulatory and/or coding regions with allele frequencies greater than 0.8. *GRK2* plays a crucial role in skeletal muscle development and differentiation, primarily by regulating key signaling pathways, such as p38MAPK and Akt. Studies in various models, including Drosophila and mice, have shown that alterations in *GRK2* levels can lead to defects in muscle differentiation, changes in muscle fiber size, and impaired muscle function. Thus, balanced *GRK2* activity is essential for proper myogenic processes and skeletal muscle growth [28]. *ASB2* functions as a negative regulator of skeletal muscle mass, influenced by the TGF-β signaling network, and follistatin-based interventions, which modulate TGF-β network activity, lead to decreased *ASB2* expression, promoting muscle hypertrophy [29].

*PDGFRA* plays a pivotal role in directing stem cell fate toward the paraxial mesoderm, which is essential for the formation of myogenic precursor cells [30,31]. *ACVR1* plays a significant role in myogenic differentiation, as demonstrated by its mutation (Acvr1R206H/+) negatively affecting this process. The mutation in fibro/adipogenic progenitors (FAPs) impairs their ability to support the myogenic potential of muscle satellite cells (MuSCs), leading to reduced fusion and formation of myofibers. Conversely, healthy FAPs can ameliorate the impaired myogenic morphology of mutant MuSCs, and the ACVR1 signaling pathway, especially in the context of the transforming growth factor beta/bone morphogenetic protein (TGFβ/BMP) pathway, is crucial, as the dysregulation of components such as *BMP2* and *Noggin* in FAP-conditioned media is associated with impaired muscle regeneration and differentiation [32]. *PGAM2*, a key glycolytic enzyme highly expressed in skeletal muscle, is critical for myogenic differentiation. Mutations in *PGAM2*, such as the K176R alteration, lead to impaired myogenic differentiation and reduced cellular glycolysis and mitochondrial function, as evidenced in CRISPR-engineered C2C12 myogenic cells [33].

*IGF1* plays a pivotal role in regulating the growth and maintenance of skeletal muscles. It promotes muscle protein synthesis through the PI3K/Akt/mTOR and PI3K/Akt/GSK3β pathways and inhibits muscle atrophy by suppressing E3 ubiquitin ligases via the PI3K/Akt pathway, thereby reducing protein degradation in the ubiquitin-proteasome system [20]. *FoxO1*, a member of the FoxO family, is a crucial transcription factor in skeletal muscle that plays a dual role in muscle growth and differentiation. While some studies suggest that *FoxO1* acts as a negative regulator of skeletal muscle differentiation, others highlight its importance in myoblast fusion [34]. Furthermore, the co-occurrence of SNPs in both the regulatory and coding regions underscores the genetic complexity of muscle development in pigs. This complexity suggests a multilayered regulation of key genes that influence muscle growth through both transcriptional and post-transcriptional mechanisms.

### Shared genes between DEGs and DEPs potentially crucial for skeletal muscle growth

In addition, our focus was extended to genes that were both DEGs and DEPs related to skeletal muscle growth, and possessed TIBP- and LWP-specific and predominant SNPs in regulatory and/or coding regions with ΔAF greater than 0.8. Concurrent changes in the levels of the gene and its protein indicate that the function of this gene is critical under certain conditions [35]. In our study, *CAPN3*, *MYOM2*, and *PGAM2* were identified in this category.

*CAPN3* is the only muscle-specific calpain that plays an important role in the promotion of calcium release from skeletal muscle fibers, calcium uptake by the sarcoplasmic reticulum, muscle formation, and muscle remodeling [36]. We discovered four synonymous, three 5′ prime UTR, and three upstream TIBP-predominant SNPs in the *CAPN3* gene, suggesting a significant role in the growth disparities between the TIBP and LWP breeds.

Five synonymous TIBP-predominant SNPs were detected in *MYOM2* in this study. *MYOM2* plays a significant role in skeletal muscle function. It is involved in the structure and integrity of the muscle sarcomere, particularly at the M-line, where it contributes to the assembly and stabilization of thick filaments. *MYOM2* interacts with other sarcomeric proteins to maintain the proper alignment and function of the sarcomere during muscle contraction. Mutations or dysregulation of *MYOM2* can affect muscle strength and may be implicated in various muscle disorders [37].

*PGAM2*, a crucial glycolytic enzyme highly expressed in skeletal muscle and essential for myogenic differentiation [33], exhibited two 5′ prime UTR premature start codon gains, six 5′ prime UTR, and one synonymous TIBP-specific and LWP-predominant SNPs. These findings underscore the potential critical role of these genes in the differences in primary fiber development between the TIBP and LWP breeds, highlighting their importance in skeletal muscle growth and development.

### Toward a holistic understanding of genetic determinants in skeletal muscle development across life stages in TIBP and LWP breeds

This study synergistically combined DEGs and DEPs from the critical stage of primary myofiber formation with genomic data to uncover potential causative genes for the differences in skeletal muscle growth between the TIBP and LWP breeds. However, it is important to recognize that skeletal muscle development is a polygenic trait exhibiting varied characteristics across different stages of life. For example, during the prenatal phase, the development of skeletal muscle primarily involves an increase in myofiber numbers, beginning with the formation of primary myofibers, followed by secondary myofibers [5]. Conversely, postnatal muscle growth is predominantly characterized by hypertrophy of existing fibers [38,39]. Therefore, a joint analysis that encompasses DEGs and DEPs from multiple developmental stages, combined with genomic data, would enable more thorough and insightful exploration. This integrative approach is crucial for identifying the genes responsible for the observed differences in skeletal muscle development between the TIBP and LWP breeds, thereby providing a complete understanding of the genetic factors influencing muscle growth across different life stages.

## CONCLUSION

In this study, we conducted an extensive investigation of SNPs present in DEGs and DEPs during the prenatal stage, focusing particularly on TIBP- and LWP-specific and predominant SNPs in regulatory and/or coding regions with marked ΔAF between the TIBP and LWP breeds. The identified DEGs and DEPs were significantly involved in the biological process of skeletal muscle growth. These included genes such as *MYF5*, *MYOF*, *ASB2*, *PDE9A*, *SDC1*, *PDGFRA*, *MYOM2*, *ACVR1*, *ZIC3*, *COL11A1*, *TGFBR1*, *EDNRA*, *TGFB2*, PDE4D, *PGAM2*, *GRK2*, *SCN4B*, *CACNA1S*, *MYL4*, *CAPN3*, and *RAP1GDS1*, as well as genes in the protein synthesis signaling pathway, such as *IGF1* and *FOXO1*. These genes have been proposed as potential causal factors that contribute to the divergence in skeletal muscle growth between the TIBP and LWP breeds. Our findings offer significant insights into the mechanisms underlying skeletal muscle development and provide valuable information for pig breeding strategies aimed at enhancing meat production.

## Figures and Tables

**Table 1:** Tibetan pigs-specific single nucleotide polymorphisms in regulatory and/or coding regions harbored by differentially expressed genes related to skeletal muscle development

Chrom	Position	SNP type	Gene name	Ref allele	Alt allele	Allele frequency of Alt allele	

						TIBP	LWP
1	240,842,119	Upstream	*TGFBR1*	C	T	0.81	0.00
2	5,208,908	Upstream	*GRK2*	T	C	0.83	0.00
2	5,208,922	Upstream	*GRK2*	T	C	0.82	0.00
2	5,208,923	Upstream	*GRK2*	G	A	0.82	0.00
3	118,010,986	3′ Prime UTR	*SDC1*	G	A	0.81	0.00
4	115,749,645	Synonymous	*COL11A1*	C	T	0.93	0.00
5	100,755,208	Synonymous	*MYF5*	C	G	0.84	0.00
7	115,249,986	Upstream	*ASB2*	A	G	0.90	0.00
7	115,250,009	Upstream	*ASB2*	A	G	0.90	0.00
7	115,250,023	Upstream	*ASB2*	G	A	0.89	0.00
7	115,250,304	Upstream	*ASB2*	A	G	0.86	0.00
7	115,250,849	Upstream	*ASB2*	A	G	0.90	0.00
7	115,250,890	Upstream	*ASB2*	A	C	0.88	0.00
7	115,250,903	Upstream	*ASB2*	T	C	0.89	0.00
8	40,994,493	Synonymous	*PDGFRA*	T	C	0.99	0.00
8	40,994,827	Synonymous	*PDGFRA*	C	T	0.87	0.00
8	41,009,677	Synonymous	*PDGFRA*	T	C	0.94	0.00
8	81,210,043	Upstream	*EDNRA*	A	G	1.00	0.00
8	81,277,681	Upstream	*EDNRA*	A	G	0.89	0.00
8	81,278,079	Upstream	*EDNRA*	A	T	0.83	0.00
9	45,468,105	Upstream	*SCN4B*	T	C	0.89	0.00
9	45,468,285	Upstream	*SCN4B*	T	C	0.90	0.00
9	45,468,683	Upstream	*SCN4B*	T	C	0.88	0.00
10	8,436,053	Downstream	*TGFB2*	T	G	0.81	0.00
10	23,568,482	Upstream	*CACNA1S*	G	C	0.95	0.00
12	16,774,572	Upstream	*MYL4*	G	A	0.93	0.00
13	205,937,377	Upstream	*PDE9A*	T	C	0.83	0.00
13	205,997,808	Upstream	*PDE9A*	T	C	0.84	0.00
13	206,000,181	5′ Prime UTR	*PDE9A*	T	C	0.86	0.00
13	206,000,259	5′ Prime UTR	*PDE9A*	T	C	0.84	0.00
13	206,033,545	Missense	*PDE9A*	T	G	0.87	0.00
13	206,034,864	3′ Prime UTR	*PDE9A*	T	C	0.89	0.00
14	10,494,0521	Upstream	*MYOF*	A	C	0.87	0.00
15	33,537,058	Splice region & intron	*MYOM2*	G	A	0.86	0.00
15	64,749,414	Upstream	*ACVR1*	T	A	0.92	0.00
15	64,750,217	3′ Prime UTR	*ACVR1*	T	A	0.92	0.00
15	64,750,438	3′ Prime UTR	*ACVR1*	T	C	0.94	0.00
15	64,756,963	3′ Prime UTR	*ACVR1*	G	A	0.93	0.00
15	64,824,052	Upstream	*ACVR1*	A	G	0.80	0.00
16	38,183,547	5′ Prime UTR	*PDE4D*	T	C	0.93	0.00
18	48,694,221	5′ Prime UTR premature start codon gain	*PGAM2*	G	A	0.83	0.00
18	48,694,249	5′ Prime UTR	*PGAM2*	A	G	0.86	0.00
18	48,694,283	5′ Prime UTR	*PGAM2*	T	A	0.82	0.00
19	112,597,017	3′ Prime UTR	*ZIC3*	A	G	0.97	0.00

SNPs, single nucleotide polymorphisms; TIBP, Tibetan pig; LWP, Large White pig; UTR, untranslated regions.

**Table 2:** Tibetan pig-specific single nucleotide polymorphisms in the regulatory region harbored by differentially expressed genes involved in the mTOR signaling pathway

Chrom	Position	SNP type	Gene name	Ref allele	Alt allele	Allele frequency of Alt allele	

						TIBP	LWP
5	81,778,318	5′ Prime UTR	*IGF1*	C	T	0.88	0.00
5	81,780,796	Upstream	*IGF1*	C	A	0.82	0.00
5	81,780,814	Upstream	*IGF1*	G	T	0.81	0.00
5	81,833,004	3′ Prime UTR	*IGF1*	A	G	0.91	0.00
5	81,833,429	3′ Prime UTR	*IGF1*	T	C	0.90	0.00
5	81,835,111	3′ Prime UTR	*IGF1*	T	C	0.81	0.00
5	81,835,227	3′ Prime UTR	*IGF1*	T	C	0.84	0.00
5	81,835,258	3′ Prime UTR	*IGF1*	A	G	0.84	0.00
5	81,835,979	3′ Prime UTR	*IGF1*	T	C	0.86	0.00
5	81,836,365	Downstream	*IGF1*	T	C	0.84	0.00
5	81,836,499	Downstream	*IGF1*	C	A	0.83	0.00
5	81,836,534	Downstream	*IGF1*	T	C	0.83	0.00
5	81,836,638	Downstream	*IGF1*	G	A	0.82	0.00
5	81,849,343	3′ Prime UTR	*IGF1*	T	A	0.93	0.00
5	81,853,114	Downstream	*IGF1*	A	T	0.91	0.00
5	81,853,171	Downstream	*IGF1*	A	G	0.94	0.00
5	81,853,172	Downstream	*IGF1*	G	A	0.94	0.00
5	81,853,917	Downstream	*IGF1*	T	A	0.88	0.00
5	81,908,700	3′ Prime UTR	*IGF1*	G	T	0.99	0.00
5	81,909,169	3′ Prime UTR	*IGF1*	C	A	0.96	0.00
5	81,910,107	Downstream	*IGF1*	A	G	0.96	0.00
11	15,319,209	3′ Prime UTR	*FOXO1*	T	C	0.94	0.00
11	15,319,295	3′ Prime UTR	*FOXO1*	A	G	0.94	0.00
11	15,319,643	3′ Prime UTR	*FOXO1*	T	C	0.94	0.00
11	15,320,069	3′ Prime UTR	*FOXO1*	G	A	0.93	0.00
11	15,320,117	3′ Prime UTR	*FOXO1*	T	C	0.94	0.00
15	48,422,881	Upstream	*EIF4EBP1*	T	C	0.85	0.00

mTOR, mechanistic target of rapamycin kinase; SNP, single nucleotide polymorphism; TIBP, Tibetan pig; LWP, Large White pig; UTR, untranslated regions.

**Table 3:** Specific and predominant single nucleotide polymorphisms in regulatory and/or coding regions harbored by shared genes among DEGs and DEPs related to skeletal muscle development

Chrom	Position	SNP type	Gene name	Ref allele	Alt allele	Allele frequency of Alt allele		ΔAF

						TIBP	LWP	
1	108,987,504	3′ Prime UTR	*TPM1*	T	C	0.91	0.11	0.81
1	128,960,185	3′ Prime UTR	*CAPN3*	G	A	0.93	0.05	0.88
1	129,012,944	Synonymous	*CAPN3*	C	A	0.87	0.03	0.85
1	129,013,136	Synonymous	*CAPN3*	C	T	0.86	0.03	0.83
1	129,013,139	Synonymous	*CAPN3*	T	C	0.86	0.03	0.83
1	129,013,184	Synonymous	*CAPN3*	G	A	0.84	0.03	0.82
1	129,013,317	5′ Prime UTR	*CAPN3*	T	C	0.89	0.03	0.86
1	129,013,319	5′ Prime UTR	*CAPN3*	C	T	0.89	0.03	0.86
1	129,013,515	Upstream	*CAPN3*	G	T	0.89	0.03	0.86
1	129,013,788	Upstream	*CAPN3*	G	T	0.85	0.03	0.83
1	129,014,494	Upstream	*CAPN3*	T	C	0.85	0.03	0.82
8	121,866,237	Upstream	*RAP1GDS1*	G	A	0.90	0.03	0.87
8	121,866,622	Upstream	*RAP1GDS1*	A	G	0.91	0.05	0.86
8	121,867,490	3′ Prime UTR	*RAP1GDS1*	T	C	0.91	0.03	0.88
8	121,868,433	3′ Prime UTR	*RAP1GDS1*	A	C	0.88	0.03	0.85
12	16,774,572	Upstream	*MYL4*	G	A	0.93	0.00	0.93
14	76,452,928	5′ Prime UTR	*MYOZ1*	A	C	0.06	0.97	0.91
15	33,537,058	Splice region & intron	*MYOM2*	G	A	0.86	0.00	0.86
15	33,569,852	Synonymous	*MYOM2*	T	C	0.86	0.05	0.81
15	33,574,636	Synonymous	*MYOM2*	T	C	0.86	0.05	0.81
15	33,577,752	Synonymous	*MYOM2*	T	C	0.86	0.05	0.81
15	33,577,812	Synonymous	*MYOM2*	C	T	0.93	0.11	0.82
15	33,606,405	Synonymous	*MYOM2*	T	C	0.87	0.05	0.82
15	33,607,063	Downstream	*MYOM2*	G	A	0.88	0.05	0.83
15	33,607,875	Downstream	*MYOM2*	C	T	0.89	0.06	0.83
18	48,694,221	5′ Prime UTR Premature start codon gain	*PGAM2*	G	A	0.83	0.00	0.83
18	48,694,249	5′ Prime UTR	*PGAM2*	A	G	0.86	0.00	0.86
18	48,694,283	5′ Prime UTR	*PGAM2*	T	A	0.82	0.00	0.82
18	48,701,904	5′ Prime UTR	*PGAM2*	C	A	0.03	0.95	0.91
18	48,709,016	5′ Prime UTR	*PGAM2*	G	A	0.04	0.97	0.93
18	48,709,414	5′ Prime UTR	*PGAM2*	C	G	0.02	0.94	0.92
18	48,710,042	5′ Prime UTR Premature start codon gain	*PGAM2*	C	T	0.04	0.84	0.81
18	48,710,267	5′ Prime UTR	*PGAM2*	C	G	0.04	0.97	0.93
18	48,712,707	Synonymous	*PGAM2*	C	A	0.04	0.94	0.90

DEGs, differentially expressed genes; DEPs, differentially expressed proteins; SNP, single nucleotide polymorphism; TIBP, Tibetan pig; LWP, Large White pig; UTR, untranslated regions; AF, allele frequency differences.

## SUPPLEMENTARY MATERIAL

**Supplementary Table S1**. Enrichment GO terms of overlapping DEGs

**Supplementary Table S2**. SNPs harbored by overlapping DEGs involved in the biological processes of skeletal muscle development

**Supplementary Table S3**. Type of SNPs harbored by DEGs related to skeletal muscle development

**Supplementary Table S4**. Enrichment KEGG pathways of overlapping DEGs

**Supplementary Table S5**. SNPs harbored by overlapping DEGs involved in mTOR signaling pathways

**Supplementary Table S6**. Type of SNPs harbored by DEGs involved in the mTOR signaling pathway

**Supplementary Table S7**. Enrichment GO terms of overlapping DEPs

**Supplementary Table S8**. SNPs harbored by overlapping DEPs involved in the biological process of skeletal muscle development

**Supplementary Table S9**. Type of SNPs harbored by DEPs related to skeeltal muscle development

**Supplementary Table S10**. Enrichment KEGG pathways of overlapping DEPs

**Supplementary Table S11**. SNPs harbored by common genes among DEGs and DEPs related to skeletal muslce development
